# ﻿A review of the *sinica* species group within the genus *Lilioceris* (Coleoptera, Chrysomelidae, Criocerinae)

**DOI:** 10.3897/zookeys.1119.87082

**Published:** 2022-08-31

**Authors:** Yuan Xu, Hongbin Liang

**Affiliations:** 1 Key Laboratory of Zoological Systematics and Evolution, Institute of Zoology, Chinese Academy of Sciences, Beijing 100101, China Institute of Zoology, Chinese Academy of Sciences Beijing China; 2 College of Life Science, University of Chinese Academy of Sciences, Beijing 100049, China University of Chinese Academy of Sciences Beijing China

**Keywords:** Distribution, genitalia, key, shining leaf beetle, taxonomy

## Abstract

A new species group of the genus *Lilioceris* Reitter, 1913 is proposed and reviewed, the *sinica* group. It includes six species: *L.gressitti* Medvedev, 1958; *L.rugata* (Baly, 1865); *L.sieversi* (Heyden, 1887); *L.sinica* (Heyden, 1887); *L.theana* (Reitter, 1898) **stat. nov.**; and *L.thibetana* (Pic, 1916). Among them, *L.theana* is resurrected as a valid species from synonymy with *L.rugata*, and is newly reported from China. Redescriptions, an identification key, figures of habitus and male and female genitalia, geographic distributions, host plants, and habitats (if known) are provided for these species.

## ﻿Introduction

*Lilioceris* Reitter, 1913 is the second largest genus of Criocerinae, includes approximately 150 species to date (Monrós 1960; Heinze and Pinsdorf 1962; Gressitt 1965; Warchałowski 2011; Bezděk and Schmitt 2017). The genus is widely distributed in tropical and subtropical parts of the world, with the highest species diversity in the Oriental Region. Species of *Lilioceris* are characterized by a more or less elongate body shape, of medium or small size (5–12 mm); the thorax is subcylindrical or subquadrate, without lateral margins, and the lateral sides constricted in middle; the tibiae has two spurs. Species of *Lilioceris* usually live in margins of forest or farmland habitats, and all life stages are associated with the host plant. Most of their host plants are from the families Smilacaceae, Dioscoreaceae, and Liliaceae (Jolivet 1988; Schmitt 1988), and a few *Lilioceris* species are economically important. *Lilioceris* is unquestionably monophyletic group within Criocerinae as shown by recent phylogenetic studies (Schmitt 1985; Matsumura et al. 2014). There are many taxonomic works focusing on regional species of the genus (e.g., Jacoby 1904, 1908; Gressitt and Kimoto 1961; Heinze and Pinsdorf 1962, 1963, 1964; Kimoto and Gressitt 1979; Tishechkin et al. 2011; Warchałowski 2011; Xu et al. 2021), but still many species are difficult to identify based on existing keys, and therefore more revisionary work is needed.

Tishechkin et al. (2011) proposed the *impressa* species group in the genus based on adults with a glabrous scutellum, flattened and short antennomeres 6–10 (wider than long), and strongly punctate elytral striae. Recently, when examining specimens of *Lilioceris* in the National Zoological Museum, Chinese Academy of Sciences, we found that some species were similar to members of the *impressa* species group, but differed in having cylindrical and longer antennomeres 6–10 (longer than wide). These species include *L.gressitti* Medvedev, 1958, *L.rugata* (Baly, 1865), *L.sieversi* (Heyden, 1887), *L.sinica* (Heyden, 1887), and *L.theana* (Reitter, 1898). We also found *L.thibetana* (Pic, 1916) to be very similar to *L.gressitti* Medvedev, and is not a member of the *impressa* group.

The primary purpose of this paper is to propose the *Liliocerissinica* species group, and properly document the species included in this new group.

## ﻿Materials and methods

The specimens from several museums and collections were examined. Collections cited in this article are indicated by the following abbreviations:

**HNHM**Hungarian Natural History Museum, Budapest, Hungary;

**IZCAS**National Zoological Museum, Institute of Zoology, Chinese Academy of Sciences, Beijing, China;

**MBSU** The Museum of Biology, Sun Yat-Sen University, Guangzhou, China;

**MCAU** The Museum of China Agricultural University, Beijing, China;

**MHU** The Museum of Hebei University, Baoding, China;

**MNHN**Museum National d’Histoire Naturelle, Paris, France;

**NHMB**Naturhistorisches Museum (Museum Frey, Tutzing), Basel, Switzerland;

**NHML**The Natural History Museum, London, UK;

**NIBR**National Institute of Biological Resources, Incheon , Korea;

**SDEI**Senckenberg Deutsches Entomologisches Institut, Germany.

Except as noted, all specimens examined are deposited in IZCAS.

Dry specimens were soaked in hot water for 1–2 h to soften the body. The abdomen was opened at its latero-apical margin and genitalia were pulled out using forceps. Genitalia were soaked in warm 10% KOH for 1 h, and dyed in Chlorazol Black E. The basal orifice of the aedeagus was injected with 100% ethanol with a micro-injector until the internal sac was fully everted. The aedeagus with its everted internal sac was photographed using a large depth-of-field 3D digital microscope (Keyence VHX–1000C), and finally edited in Photoshop. A microvial with genitalia was pinned to the specimen from which the genitalia were removed for storage.

Body length (**BL**) was measured from the anterior margin of the labrum to the apex of the elytra; body width (**BW**) was measured along the greatest elytral width (**EW**); head length (**HL**) was measured along the anterior margin of the labrum to the posterior margin of tumid gena; head width (**HW**) was measured along the widest part of the head including eyes; pronotum length (**PL**) was measured along the median line of the pronotum; pronotum width (**PW**) was measured across the widest part of the pronotum; elytra length (**EL**) was measured along the suture from the base of the scutellum to the elytral apex.

Other methods of specimen observation and preparation follow previous publications (Tishechkin et al. 2011; Li et al. 2013). Morphological terminology follows Chou et al. (1993) and Matsumura et al. (2013).

## ﻿Taxonomy of the *Liliocerissinica* species group

**Diagnosis.** Small size, length less than 9.5 mm. Head, antennae, and ventral surface black, legs black or with femora bicolored; pronotum yellow, brown, or dark brown, without metallic luster; elytra unicolored, red, brown, black, or blue, without bands or patches, without metallic luster. Antennae short, nearly 1/3 as long as body length, antennomeres 5–10 cylindrical, longer than wide, densely pubescent and punctate. Pronotum disc with punctures distinct, scattered, not aligned into rows in the middle. Scutellum lingulate, glabrous, at most pubescent along basolateral margins. Elytra with ten rows of completely punctate striae, punctures large, present at apex; intervals flat or convex at apex, without punctures. Mesosternal process short, perpendicularly connected with metasternite. Male genitalia with tegmen Y-shaped and slender, combined with second connecting membrane. Internal sac membranous, with dorsal, median, and ventral sclerites moderately sclerotized. Female genitalia with tergites 8 and 9 and sternites 8 and 9 sclerotized, posterior areas of tergite 8 and sternite 8 with dense setae, without apodemes.

Species of the *Liliocerissinica* group are similar to those of *Liliocerisimpressa* group in having glabrous scutellum, completely punctate elytral striae, and three moderately sclerotized sclerites in aedeagus. However, the most significant difference between the two groups is that antennomeres 6–10 are distinctly flattened and quadrate or even transverse (Figs 36–41) in the *impressa* group (Tishechkin et al. 2011), while obviously cylindrical (Figs 30–35) in the *sinica* group. Generally, body size in the *impressa* group (length 7.5–11.8 mm; mean 9.18 ± 0.20) is greater than that in the *sinica* group (length 6.0–9.0 mm; mean 7.13 ± 0.22).

We recognize six species belonging to *sinica* group based on examination of the type specimens and / or descriptions. As to the African *Lilioceris* treated by Heinze and Pinsdorf (1962), several species with a pronotum irregularly punctate, the elytra unicolored and strongly punctate, and the antennomeres 6–10 slightly longer than wide, probably fall into this group (e.g., *L.cafra* (Lacordaire, 1845), *L.consobrina* (Clark, 1866), *L.latipennis* (Clark, 1866), *L.lumbwensis* (Weise, 1926) *L.puncticollis* (Lacordaire, 1845), and *L livida* (Dalman, 1823)). These species will be treated when types are available to us in the future. Known host plants of the group are *Dioscorea* spp. (Dioscoreaceae).

### ﻿Key to species of the *Liliocerissinica* species group

**Table:** 

1	Lateral side of metasternite nearly glabrous, with only little pubescence occasionally near the border (Figs 13C, 14C, 17C)	**2**
–	Lateral side of metasternite with a wide or narrow strip of pubescence, extending from anterior to posterior margin (Figs 12C, 15C, 16C)	**4**
2	Pronotal disc with strong and deep punctures (Fig. 13A); punctures of elytra large and deep, intervals convex at apical 1/4 (Fig. 13D)	***L.rugata***
–	Pronotal disc with fine and shallow punctures (Figs 14A, 17A); punctures of elytra small and shallow, intervals flat at apex (Figs 14D, 17D)	**3**
3	Elytra black or blackish blue (Fig. 17D)	***L.sieversi***
–	Elytra red or yellow (Fig. 14D)	***L.thibetana***
4	Lateral side of metasternite with a narrow strip of pubescence (Fig. 12B); punctures of elytra large on basal half, diminishing posteriorly, intervals flat (Fig. 12D)	***L.gressitti***
–	Lateral side of metasternite with a wide strip of pubescence (Figs 15B, 16B); punctures of elytra large, not diminishing posteriorly, intervals convex at apical 1/4 (Figs 15D, 16D)	**5**
5	Lateral 1/4 of metasternite glabrous (Fig. 15B); lateral transverse impressions on abdominal sternites 2–5 distinct, glabrous, other area of sternite pubescent (Fig. 15C)	***L.sinica***
–	Lateral 1/4 of metasternite sparsely pubescent (Fig. 16B); lateral transverse impressions on abdominal sternites 2–5 absent, sternite wholly pubescent (Fig. 16C)	***L.theana***

#### ﻿ Lilioceris gressitti

Taxon classificationAnimaliaColeopteraChrysomelidae

Medvedev, 1958

433FB539-ED52-567D-93BD-E35DB2AB0341

Figs 1 , 12 , 18 , 24 , 30 , 42 , 43–44

gressitti Medvedev 1958: 111 (China, Prov. Yunnan, holotype, gender ?).

##### Type material examined.

1 holotype (NHMB, photo), China, Prov. Yunnan, Vallis flumin Soling-ho / Liliocerisgressitti m, L. N. Medvedev det. 1957, holotype / Type.

**Figures 1–3.**
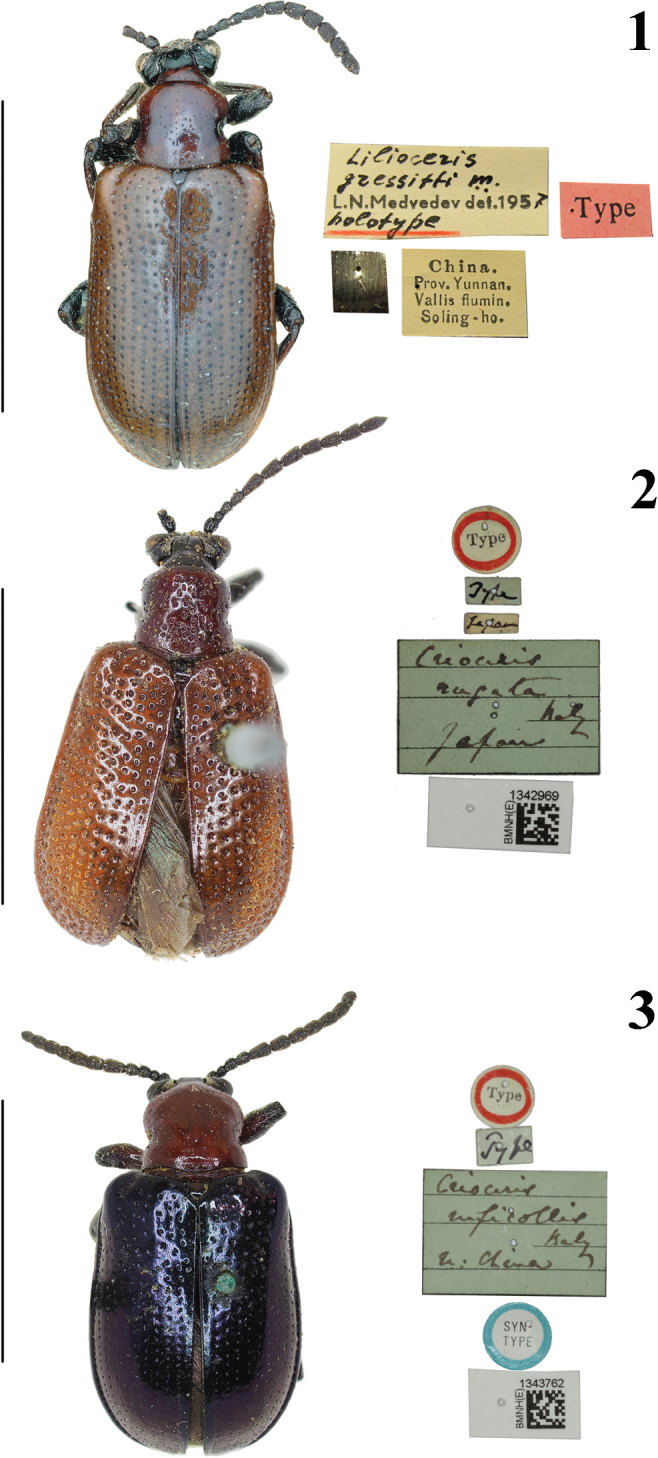
Habitus of *Lilioceris* spp. **1***L.gressitti*, type, China (Yunnan), photographed by Christoph Germann **2***L.rugata*, type, Japan, photographed by Hongbin Liang **3***L.ruficollis*, type, north of China, photographed by Hongbin Liang. Scale bars: 5.0 mm.

##### Other material examined.

Total 24 specimens. **China: Yunnan**: 1♂, Kunming, 1941.V.23 / Liliocerisgressitti Medvedev, Peiyu Yu Det.; 1♂, Kunming, 1942.VI.27; 1♂1♀, Kunming, Zhujie Temple / 1958.IX.10; 1♂1♀, Yongsheng, Liude, 2400 m / 1984.VII.18, Shuyong Wang coll.; 1♂, Tengchong, Dahaoping, Hao Huang, 2005.VI; 1♂, Xishuangbanna, Mengzhe, 1750 m / 1958.VI.25, Fuji Pu coll.; 1♂, Xishuangbanna, Menghai, 1200–1600 m / 1958.VII.22, Fuji Pu coll.; 1♀, Kunming, Anning / 1980.VIII.6, 1900 m / Liliocerisgressitti Medvedev, Peiyu Yu Det.; 2♀, Xishuangbanna, Mengsong, 1600 m / 1958.VII.25, Leyi Zheng coll.; 1♀, Xishuangbanna, Menghai, 1200–1600 m / 1958.VII.22, Shuyong Wang coll.; 1♀, Lancang, 1100 m / 1957.VIII.8, Shuyong Wang coll.; Kunming, suburb, 1900 m, 1956.II.16, Panfilov coll.; 3♂5♀, Wuding, Chadian, Changji Road, 25.74144°N, 102.30336°E / 2296 m, 2020.VII.11 D1, Yuan Xu & Neng Zhang coll.; 1♂2♀ (MHU), Puer, Laiyang River, 2007.VII.28, Guodong Ren, Wenjun Hou & Yachai Li coll.; 1♂ (MHU), Lvchun, 2004.VII.27, Jing Li & Caixia Yuan coll.; 1♂ (MCAU), Kunming, 1946.V; 1♂ (MCAU), Kunming, Xi Shan, V.16; 1♂ (MCAU), Kunming, 1947; **Sichuan**: 1♀, Xiangcheng, 2900–3200 m / 1982.VI.28, Shuyong Wang coll.; **Guizhou**: 1♂ (MHU), Yinjiang, Fanjing Shan, 2010.VIII.19–21, Yiping Niu & Yong Zhou coll.

##### Diagnosis.

Femora bicolored, black with brownish red middle; pronotum disc with fine punctures; elytral punctures large on basal half, diminishing posteriorly; lateral side of metasternite with a narrow strip of pubescence; abdominal sternites with a row pubescence, interrupted in the middle, lateral transverse impressions present on sternites 2–5, with sparse pubescence outside the impressions.

##### Redescription.

BL = 6.0–7.0 mm, BW = 3.0–3.5 mm. The front part of the head, antennae, ventral surface black; occiput, pronotum, scutellum and elytra brownish red, femora bicolored, brownish red with apex black.

***Head*** (Fig. 1). HL/HW = 1.2–1.5; vertex with a shallow groove in the middle, punctate and setose laterally; frontoclypeal area triangular, disc with fine punctures and sparse setae; labrum transverse, with sparse setae; antennomeres 5–10 slightly longer than wide (Fig. 30).

***Pronotum*** (Figs 1, 12A). PW / HW = 1.1–1.3, PL / PW = 1.1–1.2; anterior angle slightly protruding; posterior angle not protruding; sides distinctly constricted in the middle; middle of disc with fine and scattered punctures; anterior and posterior transverse impression indistinct, basal transverse groove shallow.

***Elytra*** (Figs 1, 12D). EL/EW = 1.2–1.4; sutural angle rounded; humeri protruding, humeral groove distinct, basal transverse impression indistinct; scutellary striole composed of 3–5 punctures; strial punctures large at base, diminishing posteriorly; intervals flat, at most convex at extremity of intervals 9 and 10; epipleura raised, with a row of fine punctures.

***Mesosternite pubescent***. Lateral side of metasternite with narrow strip of pubescence, extending from anterior margin to lateroposterior corner (Fig. 12B); metepisternum densely pubescent.

***Abdominal sternites*** with a row of pubescence, interrupted in the middle; lateral transverse impressions present on sternites 2–5, area outside the impression densely pubescent (Fig. 12C).

***Legs*** slender; tibiae with dense punctures and pubescence; femora with dense pubescence on dorsal surface, with sparse pubescence on ventral surface.

***Male genitalia*** (Fig. 18A–D). Median foramen occupying 1/5 length of median lobe (Fig. 18A); apex rounded (Fig. 18B); basal piece of the tegmen triangular, lateral lobes strongly sclerotized; posterior part of dorsal sclerite in dorsal view more or less parallel-sided, slightly narrowed at apex (Fig. 18C, D).

***Female reproductive organs*** (Fig. 24A–C). Spiculum gastrale short, X-shaped, distal part strongly widened, apical margin straight; ovipositor with dense setae, distal part of the ovipositor cylindrical, short, with a small protuberance; spermatheca greatly convoluted.

##### Distribution.

China (Yunnan, Sichuan, Guizhou).

##### Host plant and habitat.

(Figs 43, 44) One collecting locality of *L.gressitti* in Wuding county of Yunnan province is situated in subtropical area. This species fed on *Dioscorea* sp. (Dioscoreaceae) according to observations of the first author (XY) in Yunnan (Fig. 44). The vegetation is subtropical evergreen forest. The climate is characterized by distinct rainy summer and dry winter, annual temperature generally ranges from 6 °C to 22 °C. The forests are composed of tall trees, woody vines, and epiphytes. The host plant *Dioscorea* sp. shares its habitat with other plants such as *Pinusyunnanensis* (Pinaceae), *Alnus* sp. (Betulaceae), *Eucalyptus* sp. (Myrtaceae), *Adiantum* sp. (Pteridaceae), *Abelia* sp. (Caprifoliaceae), *Ageratina* sp. (Asteraceae), *Artemisia* sp. (Asteraceae), and *Ficus* sp. (Moraceae). *Liliocerisfouana* are collected together with this species.

##### Remarks.

Medvedev (1958) indicated that *L.gressitti* was similar to *L.rugata* (Baly, 1865), especially to *L.rugatasparsipunctata* Medvedev, 1958 (synonymized with *L.sinica* by Gressitt and Kimoto 1961), but differed by the smaller and narrower body, finer punctures on the pronotum, and less strong punctures on the elytra. In addition, it differs from *L.sinica* in the abdominal sternites having less pubescence.

#### ﻿ Lilioceris rugata

Taxon classificationAnimaliaColeopteraChrysomelidae

(Baly, 1865)

48F71445-129F-5BC5-9D38-BC8579D55CED

Figs 2 , 13 , 19 , 25 , 31 , 42 , 45

rugata Baly, 1865: 154 (Japan, syntype, gender ?). (Crioceris). Chûjô 1941: 453 (Lilioceris).

##### Type material examined.

1 type (NHML, photo), Type / Type / Japan / Criocerisrugata Baly, Japan / BMNH (E) 1342969.

##### Other material examined.

Total 10 specimens. 1♂1♀, Museum Paris, Nippon Moyen, env de Tokyo et alpes de Nikko, J. Harmand, 1901; 1♀, Karisnmi, 1932.VII.23; 1♂, Mont Takao, Pr. Hachigji, Japon: 1911.V.28, Edme Gallois / Lilioceris Rugata (Baly), Peiyu Yu Det.; 1♂, Kyoto, 1931.I.18, K. Eki; 1♂, Kibune, Kyoto, 1931.VI.14, K. Eki / Criocerisrugata Baly, det by S. Yie; 1♂, Japan, G. Lewis, 1910–320 / Criocerisrugata Baly, P. M. Hammond det. 1980; 1♂2♀, Karuizawa, 1907.IX.27 / Liliocerisrugata, det. Peiyu Yu.

##### Diagnosis.

Femora black; pronotum disc with large punctures; elytral punctures strong and not diminishing posteriorly, intervals convex apically; lateral side of metasternite nearly glabrous, without strip of pubescence; abdominal sternites smooth.

##### Redescription.

BL = 6.9–8.0 mm, BW = 3.2–3.8 mm. Head, legs, and ventral surfaces black, pronotum, scutellum, and elytra brownish red.

***Head*** (Fig. 2). HL/HW = 1.3–1.4; vertex with groove and fovea in the middle, punctate and setose laterally; frontoclypeal area triangular, disc with fine punctures and sparse setae; labrum transverse, with long setae on both apical angles; antennomeres 5–7 nearly 1.5 times as long as wide, 8–10 slightly longer than width (Fig. 31).

***Pronotum*** (Fig. 13A). PW / HW = 1.0–1.2, PL / PW = 1.0–1.3; anterior angle slightly protruding; posterior angle not protruding; sides slightly constricted in the middle; middle of disc with large and deep punctures; anterior and posterior transverse impressions indistinct, basal transverse groove shallow.

***Elytra*** (Fig. 13D). EL/EW = 1.5–1.7; sutural angle rounded; humeri protruding, humeral groove shallow, basal transverse impression indistinct; scutellary striole composed of 4–7 punctures; strial punctures large, not diminishing posteriorly, intervals convex at apical 1/4; epipleura raised, with row of fine punctures.

***Mesosternite pubescent***. Lateral side of metasternite nearly glabrous, with little pubescence along anterior and posterior margins (Fig. 13B); metepisternum densely pubescent.

***Abdominal sternites*** nearly smooth (Fig. 13C).

***Legs*** slender; tibiae with dense punctures and pubescence; femora with dense pubescence on the dorsal surface, with sparse pubescence on the ventral surface.

***Male genitalia*** (Fig. 19A–D). Median foramen occupying 1/5 length of median lobe (Fig. 19A); apex rounded (Fig. 19B); basal piece of the tegmen triangular, lateral lobes weakly sclerotized; posterior part of dorsal sclerite in dorsal view widely rounded, directed laterally (Fig. 19C, D).

***Female reproductive organs*** (Fig. 25A–C). Spiculum gastrale short, X-shaped, distal part strongly widened, apical margin straight; ovipositor with dense setae, distal part of ovipositor cylindrical and short, with a small protuberance; spermatheca small and greatly convoluted.

##### Host plant and habitat.

This species feeds on *Dioscoreajaponica* and *D.tokoro* (Kimoto, 1964). The habitat is unknown.

##### Distribution.

Japan.

##### Remarks.

This species is similar to *L.sinica*, but differs from the latter by having its pronotal disc with strong and deep punctures, metasternite and abdominal sternites nearly smooth (in *L.sinica*, pronotal disc with fine and shallow punctures, lateral side of metasternite with a wide strip of pubescence, and abdominal sternites densely pubescent except the glabrous abdominal transverse impressions). In addition, the genitalia of *L.rugata* differ from those of *L.sinica* by posterior part of dorsal sclerite in dorsal view widely rounded, directed laterally (in *L.sinica*, posterior part of dorsal sclerite in dorsal view slightly narrowed at apex, more or less parallel-sided). Chûjô (1941) synonymized *L.sinica* with *L.rugata*, which is not justified in the light of the present study.

*Liliocerisrugata* is widely distributed in Japan, obviously isolated geographically from other species in China, Russian and Korea. The records of this species from Russia and Korea are questionable. We found some photographs identified as *L.rugata* on websites from Russia (https://www.zin.ru/animalia/coleoptera/eng/lilrugkm.htm) and Korea (https://blog.naver.com/onegunah/110021296278) that are actually *L.theana*. Cho and An (2020) recorded nine specimens of *L.rugata* collected from South Korea. They are probably also *L.theana* (see Cho and An 2020: 7, fig. 13). The materials of *L.rugata* from Russia and Korea need more study.

#### ﻿ Lilioceris sieversi

Taxon classificationAnimaliaColeopteraChrysomelidae

(Heyden, 1887a)

352EAE51-5E2C-511B-9A97-7D28BCEF08F0

Figs 3 , 14 , 20 , 26 , 32 , 42 , 46

sieversi Heyden, 1887: 271 (China, Mun. Pecking). (Crioceris). Medvedev 1958: 108 (Lilioceris).
ruficollis Baly, 1865: 155 (N. China, syntype, gender ?) (Crioceris). [homonym of Criocerisruficollis Fabricius, 1787].
ruficollis White, 1981: 41 [replacement name of Criocerisruficollis Baly, 1865].

##### Type material examined.

1 syntype of *Criocerisruficollis* (NHML, photo), Criocerisruficollis Baly, N China / SYN-TYPE / BMNH (E) 1343762.

##### Other material examined.

Total 64 specimens. **China**: Heilongjiang: 2♂3♀, Harbin, Ertsentientze, Manchuria, 1941.VI.15; **Jilin**: 1♂1♀, Ma-an Shan / Liliocerisruficollis Baly, Peiyu Yu Det.; **Beijing**: 1♀, Badaling, 700 m / 1962.VI.30, Chunguang Wang coll. / Liliocerisruficollis (Baly), Peiyu Yu Det.; 1♀1♂, Badaling, 570 m / 1962.VIII.23, Shuyong Wang coll.; 2♀1♂, Badaling, 570 m / 1962.VIII.23, Shengqiao Jiang coll.; 1♀1♂, Badaling, 570 m / 1962.IX.6, Shuyong Wang coll.; 1♀, Shangfang Shan, 400 m / 1961.VII.18, Xuezhong Zhang coll.; 2♀, Sanpu / 1974.VII.18, Shengqiao Jiang coll.; 3♂, Sanpu / 1973.VIII.23, Shengqiao Jiang coll.; 1♂, Sanpu / 1980.VI.12, Jiang coll. / Liliocerisruficollis (Baly); 1♂, Sanpu / 1973.VIII.23, Shengqiao Jiang coll. / Dioscoreanipponica Makino; 1♂, Badaling, 700 m / 1962.VI.29, Chunguang Wang coll.; 1♂, Shangfang Shan, 400 m / 1961.VII.14, Shuyong Wang coll.; 1♂, Mentougou, Yanchi, 301 m, 40.00237°N, 115.80577°E, 2021.VII.8, Yuan Xu, Yuyao Qin & Hongbin Liang coll.; 1♂, Changping, Baiyanggou, 301 m, 40.23828°N, 115.96238°E, 2021.VII.8, Yuan Xu, Yuyao Qin & Hongbin Liang coll.; 1♀, Shangfang Shan, Shengshuiyu, Yunxia Shanzhuang, 566 m, 39.65727°N, 115.78220°E, 2021.07.16, Yuan Xu, Yuyao Qin & Hongbin Liang coll.; 1♀1♂, Mentougou, Yanchi, 301 m, 40.00237°N, 115.80577°E, 2021.08.26, Hongbin Liang coll.; 3♀1♂, Mentougou, Zhaitang, Malan forest farm, 2021.VI.14, Meiying Lin coll.; 3♀3♂, Mentougou, Zhaitang, Cenfu, 2021.06.12, Meiying Lin coll.; 1♀, Mentougou, Wangping, Guacaodi Scenic Area, 2021.VIII.12, Yong Wang coll.; **Hebei**: 1♀, Chahar / Chahar, Yangkiaping / 1937.VII.6, O. Piel coll. / Liliocerisruficollis (Baly), Peiyu Yu det.; 2♀, Chahar / Chahar Yangkiaping / 1937.VII.3, O. Piel coll.; 1♀, Chahar / Chahar, Yangkiaping / 1937.VII.5, O. Piel coll.; 1♂, Chahar / Chahar, Yangkiaping / 1937.VII.6, O. Piel coll.; 1♂, Xinglong, Taqian, 700 m / 1963.VII.3, Shengqiao Jiang coll.; **Hubei**: 2♂, Shennongjia, Zongluo, 900 m, 1981.VI.18, Yinheng Han coll.; **Shaanxi**: 1♀, 1936.6.9; **Zhejiang**: 2♂, Tianmu Shan, 1931.5.30; **Guizhou**: 1♂, 1910; **Jiangxi**: 1♂, Tonggu, 500 m / 1973.IV.24 / Liliocerisruficollis (Baly), Peiyu Yu det.; **Fujian**: 1♀, Wuyi Shan, 1982.6.26, Fan Jiang coll. / Liliocerisruficollis (Baly), Peiyu Yu det.; 1♀, Fuzhou / 1955.IV.21; 1♀, Fuzhou / 1955.IV.23; 1♀, Fujian; 1♀, Fuzhou, 1955.VIII.10; 1♀1♂, Fu-an, Shizitou, 1946.V.9; 1♀, Fu-an, Baisha, 1946.V.3–25; 1♂, Fuding, 1946.V; 1♂, Fuzhou / 1955.IV.23.

##### Diagnosis.

Pronotum brownish red, elytra black or dark blue, femora black; pronotum disc with fine punctures; elytral punctures large on basal half, diminishing posteriorly; metasternite almost glabrous; abdominal sternites have a row pubescence and the rest of area nearly smooth, transverse impressions present on sternites 2–5, area outside the impression with sparse pubescence.

##### Redescription.

BL = 6.5–8.5 mm, BW = 3.5–4.5 mm. Front part of head, antennae, legs, ventral surface black; occiput, pronotum brownish red, elytra dark blue or black; scutellum black slightly with brownish red.

***Head*** (Fig. 3). HL/HW = 1.3–1.5; vertex without or with an indistinct groove in the middle, finely punctate and setose laterally; frontoclypeal area triangular, disc with dense punctures and setae; labrum transverse, with sparse setae; antennomeres 5–10 slightly longer than wide (Fig. 32).

***Pronotum*** (Fig. 14A). PW / HW = 1.0–1.1, PL / PW = 1.2–1.3; anterior and posterior angle not protruding; sides constricted in the middle; middle of disc with fine and scattered punctures; anterior and posterior transverse impression indistinct, basal transverse groove shallow.

***Elytra*** (Fig. 14D). EL/EW = 1.5–1.8; sutural angle rounded; humeri protruding, humeral groove distinct, basal transverse impression indistinct; scutellary striole composed of 5–8 punctures; strial punctures large at base, diminishing posteriorly; intervals flat; epipleura raised, with row of fine punctures.

***Mesosternite pubescent*.** metasternite almost glabrous, only with sparse pubescence (Fig. 14B); metepisternum densely pubescent.

***Abdominal sternites*** with a distinct row of pubescence, sparse in the middle; lateral transverse impressions present on sternites 2–5, area outside the impression sparsely pubescent (Fig. 14C).

***Legs*** slender; tibiae with dense punctures and pubescence; femora with dense pubescence on the dorsal surface, with sparse pubescence on the ventral surface.

***Male genitalia*** (Fig. 20A–D). Median foramen occupying 1/4 length of median lobe (Fig. 20A); apex triangular (Fig. 20B); basal piece of tegmen triangular, lateral lobes weakly sclerotized; posterior part of dorsal sclerite in lateral view curved, directed ventrally, narrowed at apex in dorsal view (Fig. 20C, D).

***Female reproductive organs*** (Fig. 26A–C). Spiculum gastrale short, Y-shaped, distal part widened; ovipositor with dense setae, distal part of ovipositor cylindrical, short, with small protuberance; spermatheca slightly convoluted.

##### Distribution.

China (Heilongjiang, Jilin, Beijing, Hebei, Shaanxi, Hubei, Zhejiang, Guizhou, Jiangxi, Fujian); Korea (Park et al. 2012; Cho and An 2020).

##### Host plant and habitat.

This species feeds on *Dioscoreanipponica* and *D.polystachya* in Beijing according to our observations; in addition, *D.septemioba*, *D.batatas*, and *D.japonica* are also its hosts (Park et al. 2012).

One collecting locality of it in Beijing (Fig. 46) is situated at the north temperate zone. The climate is a temperate monsoon climate, with hot and rainy summers, and cold and dry winters with an average temperature below 0 °C. Affected by the climate, temperate deciduous broad-leaved forests grow here. The host plant *Dioscoreapolystachya* shares a habitat with other plants such as *Koelreuteriapaniculata* (Sapindaceae), *Menispermumdauricum* (Menispermaceae), Vitexnegundovar.heterophylla (Lamiaceae), *Populus* sp. (Salicaceae), *Ulmuspumila* (Ulmaceae), *Humulusscandens* (Cannabaceae), *Persicaria* sp. (Polygonaceae) and others.

##### Remarks.

This species is unique in *sinica* group for its dark blue or black elytra, aedeagus with an acute apex, and the dorsal sclerites curved in lateral view. The color of ventral side and the femora of this species is variable: specimens from northern China are completely black, while those from southern China are black with brownish red.

#### ﻿ Lilioceris sinica

Taxon classificationAnimaliaColeopteraChrysomelidae

(Heyden, 1887b)

5253247D-841E-5B8F-B277-CBB096C2219B

Figs 6 , 7 , 15 , 21 , 27 , 33 , 42 , 47–50

sinica Heyden, 1887: 270 (China, Mun. Pecking, syntype, gender ?) (Crioceris). Medvedev 1958: 112 (Lilioceris).
chinensis Jacoby, 1888: 340 (China, Pref. Kiukiang, syntype, gender ?) (Crioceris) [synonymized by Gressitt and Kimoto 1961: 58].
rugata sparsipunctata Medvedev, 1958: 111 (China, Mount. Tienmuschan, holotype, gender ?) [synonymized by Gressitt and Kimoto 1961: 58].

##### Type material examined.

1 syntype of *Liliocerissinica* (SDEI, photo), Pecking, Staudgr. 1885 / crioceris 2 / Syntypus / SDEIColeoptera # 300896; 1 syntype of *Liliocerischinensis* (NHML, photo), Syntype / Kiukiang / Jacoby coll. 1909-28a / BMNH (E) 1343930; Holotype of *Liliocerisrugatasparsipunctata* (NHML, photo), Tienmuschan, N.W. China Rtt. / Liliocerisrugata sbsp. sparsipunctata m. L N. Medvedev det. 1957 holotype / Type.

##### Other material examined.

Total 208 specimens (gender undetermined). **Beijing**: 2, Fangshan, 400 m, 1961.VI.17–18 / Shuyong Wang coll.; 4, Haidian, Xiang Shan, Yingtaogou, 40.01027°N, 116.19609°E / 131 m, 2021.VII.16, Yuan Xu, Yuyao Qin & Hongbin Liang coll.; 3, Fangshan, Shengshuiyu, Yunxia Shanzhuang, 565 m, 39.65727°N, 115.78220°E, 2021.VII.16, Yuan Xu, Yuyao Qin & Hongbin Liang coll.; 1, Miyun, Shicheng, Wangzhuang, 2020.VIII.9, Pengchang Yan coll.; 1 (MCAU), Xiang Shan, 1962.VII.12, Zhenping Zhu coll.; 1 (MCAU), Ming Tombs, 1956.VII.24, Jikun Yang coll.; **Shaanxi**: 1, 1936.V.3; 1, Liuba, Miaotaizi, 1470 m / 1999.VII.1, Chaodong Zhu coll.; 1 (MCAU), Zhongnan Shan, Taiyigong, 1956.VI.26, Jikun Yang coll.; **Shandong**: 6, Jinan; **Jiangsu**: 1, Nanjing Tangshan, 1935.V.8; 5, cemetery of Chen, 1935.IV.7–V.27; 1, Nanjing, 1923.V.16; **Henan**: 1, Xinyang, Shangcheng, Huangbai Shan, 31.3816°N, 115.3017°E / 850 m, 2020.VII.13, Pingzhou Zhu coll.; 1, Xinyang, Xinxian, Jinlan Shan, 31.6213°N, 114.7980°E / 657 m, 2020.VII.9, Lihao Zheng coll.; 1, Tongbai, Tongbai Shan, 32.3560°N, 113.3428°E / 416 m, 2020.VII.25, Lihao Zheng coll.; 5, Xinyang, Tanjiahe, 31.8683°N, 113.9382°E / 285 m, 2020.VII.7, Pingzhou Zhu coll.; 2, Xinyang, Jigong Shan, 31.8011°N, 114.0745°E / 730 m, 2020.VII.4, Pingzhou Zhu coll.; **Hubei**: 1, Shennongjia Songbai Town, 900–1200 m / 1981.V.23, Yinheng Han coll.; **Hunan**: 1, Chengbu, Dankou, 2018.05.07, Kaiqin Li coll.; **Zhejiang**: 7, Tianmu Shan, 1936.VI.9–VII.23; 1, Tianmu Shan, 1937.V.11; 1, Tianmu Shan, 1937.VIII.14; 1, Tianmu Shan, 1932.V.8; 7, Mogan Shan, 1936.IV.30–V.29; 3, Mogan Shan, 1935.V.21–VI.7; 1, Mogan Shan, 1937.VI.9; 11, Zhoushan, 1931.V.3–VI.3; 1, Zhoushan, 1923.VII.7; 4, Zhoushan, 1935.VI.12–19; 2, Zhoushan, 1934.VI.28; 2, Hangzhou, 1933.V.18–19; 1, Hangzhou, 1925; 1, Hangzhou, 1954.VI.12; 1, Taizhou, 1924.IV.30; 1, Gushan, 1933.V.23; 1, Hangzhou, West Lake, 1931.V.3; 1 (MCAU), Tianmu Shan, Chanyuan Temple, 1957.VII.1, Fasheng Li coll.; **Jiangxi**: 2, Tonggu, Taiyangling, 1974.XI.25; **Fujian**: 45, Chongan, Xingcun, Sangang, 740–900 m / 1960.V.14–VIII.24, Yiran Zhang, Chenglin Ma, Fuji Pu & Shengqiao Jiang coll.; 1, Chongan, Xingcun, San-gang, 720 m / 1973.VI.9, Peiyu Yu coll.; 8, Chongan, Xingcun, Qili Bridge, 840–870 m / 1960.V.25–VI.25, Shengqiao Jiang, Fuji Pu coll.; 14, Chongan, Xingcun, Tongmuguan, 800–1150 m / 1960.V.15–VII.10, Shengqiao Jiang, Yiran Zhang & Chenglin Ma coll.; 12, Jianyang, Dazhulan, Xianfengling, 950–1170 m / 1960.V.2–VII.5, Chenglin Ma, Yiran Zhang, Fuji Pu; 9, Jianyang, Huangkeng, Aotou, 680–950 m / 1960.IV.26–VIII.8, Fuji Pu & Yiran Zhang coll.; 4, Chongan, Xingcun, Tongmuguan, Guanping, 800–1000 m / 1960.V.30–VIII.13, Shengqiao Jiang & Fuji Pu coll.; 4, Chongan, Xingcun, Longdu, 580–800 m / 1960.V.19–VI.5, Shengqiao Jiang & Yong Zuo coll.; 1, Jianyang, Huangkeng, Dazhulan, 900–1170 m, 1960.VII.24, Jiang Shengqiao; 1, Jianyang, Huangkeng, Dazhulan, 900 m / 1973.VI.6, Peiyu Yu coll.; 1, Chongan, Chengguan, 240 m / 1960.IX.19, Yiran Zhang coll.; 1, Chongan, Wuyishan Sanatorium, 175–300 m / 1960.VII.3, Fuji Pu coll.; 2, Jianyang, Huangkeng, Guilin, 270 m / 1960.IV.11, Yiran Zhang coll.; 1, Dazhulan, 1948.VL.20; 2, Fujian; Chongan, Xingcun, Shili Factory, 840 m / 1960.V.25, Shengqiao Jiang coll.;1, Chongan, Xingcun, Guadun, 900–1160 m / 1960.VI.8, Chenglin Ma coll.; 1, Chongan, Xingcun, Sangang, 700 m / 1982.VI.8, Juanjie Tan coll.; **Guangxi**: 1, Ziyuan, 1976.VII.14, Baolin Zhang coll.; 3, Guilin, 1952.IV.19–XII.8; 2, Guilin, Yan Shan, 1953.IV.24–V.12; 1, Yan Shan, 1952.XI.24; 1, Yangshuo; 1, Yao Shan, 1938.V.6; 1, Baishou, 1952.VI.28; **Sichuan**: 4, Luding, Moxi, 1500 m / 1983.VL.17–20, Shuyong Wang; 1, Xiangcheng, 2900–3200 m, 1982.VI.28, Shuyong Wang coll.; **Guizhou**: 7, Huaxi, 2000.VI.8; 1, Bazhai, 1930.VII.22;3, Guizhou; 1 (MBSU), Kweichow. SW. China, Kweiyang, alt. 1000 meters. 1940.VII.11, J. L. Gressitt / chinensis / Criocerischinensis Jac., J. L. Gressitt det. 1940 / Liliocerissinica (Heyden), det. Jianguo Long / En–077357; **Yunnan**: 1, Yongsheng, Liude, 2100 m / 1984.VII.18, Shuyong Wang coll.; 1 (MCAU), Kunming, 1946.V.

##### Diagnosis.

Femora bicolored, black with brownish red middle; pronotum disc with fine punctures; elytral punctures strong, not diminishing posteriorly, intervals convex at apical 1/4; lateral side of metasternite with a wide strip of pubescence; abdominal transverse impressions present on lateral area of sternites 2–5, glabrous, other area of sternite pubescent.

##### Redescription.

BL = 6.2–9.0 mm, BW = 3.0–4.5 mm. The front part of the head, antennae, ventral surface black; occiput, pronotum, scutellum and elytra brownish red, femora bicolored, brownish red with apex black.

***Head*** (Fig. 6). HL/HW = 1.1–1.2; vertex with a shallow groove in the middle, punctate and setose laterally; frontoclypeal area triangular, disc with dense punctures and setae; labrum transverse, with long setae on both apical angles; antennomeres 5–10 slightly longer than their widths (Fig. 33).

***Pronotum*** (Fig. 15B). PW / HW = 0.9–1.1, PL / PW = 1.0–1.1; anterior angle slightly protruding; posterior angle not protruding; sides distinctly constricted in the middle; middle of disc with fine punctures; anterior and posterior transverse impression indistinct, basal transverse groove shallow.

***Elytra*** (Fig. 15D). EL/EW = 1.4–1.6; sutural angle rounded; humeri protruding, humeral groove shallow, basal transverse impression indistinct; scutellary striole composed of 4–7 punctures; strial punctures large, not diminishing posteriorly, intervals convex at apical 1/4; epipleura raised, with a row of fine punctures.

***Mesosternite pubescent***; lateral side of metasternite with wide strip of pubescence, extending from anterior to posterior margin, lateral 1/4 near metepisternum glabrous (Fig. 15B); metepisternum densely pubescent.

Lateral transverse impressions present on abdominal sternites 2–5, other area of sternite densely pubescent (Fig. 15C).

***Legs*** slender; tibiae with dense punctures pubescence; femora with dense pubescence on the dorsal surface, with sparse pubescence on the ventral surface.

***Male genitalia*** (Fig. 21A–D). Median foramen occupying 1/5 length of median lobe (Fig. 21A); apex rounded (Fig. 21B); basal piece of the tegmen triangular, relatively broad, lateral lobes weakly sclerotized; posterior part of dorsal sclerite in dorsal view more or less parallel-sided, slightly narrowed at apex (Fig. 21C, D).

***Female reproductive organs*** (Fig. 27A–C). Spiculum gastrale long, Y-shaped, distal part slightly widened, apical margin rounded; ovipositor with dense setae, distal part of the ovipositor cylindrical, short, with a small protuberance; spermatheca simply convoluted.

##### Distribution.

Beijing, Shandong, Shaanxi, Henan, Jiangsu, Hubei, Hunan, Zhejiang, Jiangxi, Fujian, Guangxi, Yunnan, Sichuan, Guizhou; Korea (Cho and An 2020).

##### Host plant and habitat.

(Figs 47–50) This species feeds on *Dioscoreapolystachya* according to our field observation in Beijing (Fig. 49).

This species lives on elevations from 131 to 3200 m. One collecting locality of *L.sinica* in Beijing (Fig. 50) is situated at the north temperate zone. The climate here is a temperate monsoon climate, with hot and rainy summers, and cold and dry winters with an average temperature below 0 °C. Affected by the climate, temperate deciduous broad-leaved forests grow here. The host plant *Dioscoreapolystachya* shares habitat with other plants such as *Metasequoiaglyptostroboides* (Cupressaceae), *Juniperuschinensis* (Cupressaceae), Pinus tabuliformis (Pinaceae), *Syringaoblata* (Oleaceae), *Morusalba* (Moraceae), Vitexnegundovar.heterophylla (Lamiaceae), *Inulajaponica* (Asteraceae), *Polygonumaviculare* (Polygonaceae), *Potentillachinensis* (Rosaceae) and *Oxaliscorniculate* (Oxalidaceae).

##### Remarks.

**Figures 4, 5.**
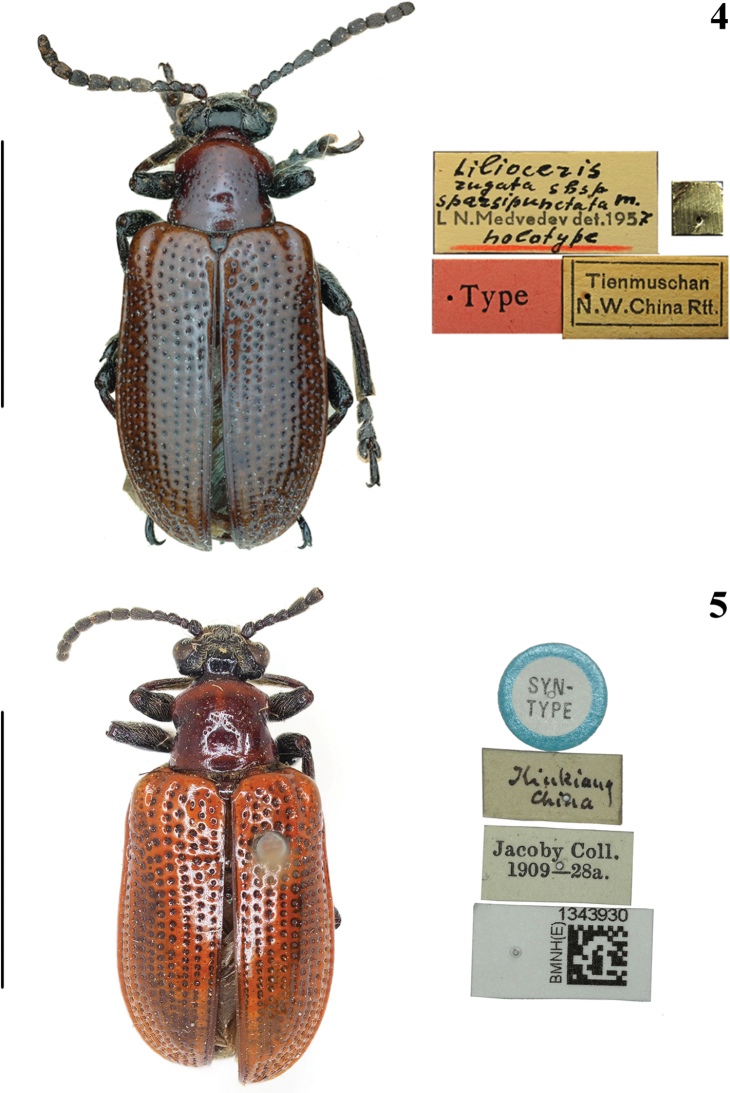
Habitus of *Lilioceris* spp. **4***L.rugatasparsipunctata*, type, China (Tienmuschan = Tianmu Shan), photographed by Christoph Germann **5***L.chinensis*, syntype, China (Kiukiang = Jiujiang), photographed by Hongbin Liang. Scale bars: 5.0 mm.

*Liliocerisrugatasparsipunctata* Medvedev, 1958 was described from Zhejiang and *Liliocerischinensis* (Jacoby 1888) was described from Jiangxi. Gressitt and Kimoto (1961: 58) synonymized them with *L.sinica*. We compared the types (Figs 4, 5) and agree with their treatment.

*Liliocerisjakobi* (White 1981) was originally described as *Liliocerisminima* by Jakob (1961) from Zhejiang and Fujian (White 1981). This species is similar to *L.chinensis* according to original literature (Jakob 1961), but it has a smooth pronotum, so should not belong to the *sinica* group. Unfortunately, the status of this species is unclear because we could not locate the type depository.

#### ﻿ Lilioceris theana

Taxon classificationAnimaliaColeopteraChrysomelidae

(Reitter, 1898)

3F432D1B-6F9A-5608-B5A7-CAD1DAABD29A

Figs 8 , 9 , 16 , 22 , 28 , 34 , 42 , 51–54

theana Reitter, 1898: 22 (Russia, Sibiria, holotype, gender ?). (Crioceris). Chûjô 1941: 453 (Lilioceris).

##### Type material examined.

***Holotype*** (HNHM, photo), Sibirien, Reitter Leder / Siberia Chabarowba, leg. Graeser / Cr. theana m. 1897 / Holotypus, 1898, Crioceris theama [mis-spelling of theana], Reitter / Coll. Reitter.

##### Other material examined.

Total 92 specimens. **China**: **Heilongjiang**: 1♀ Harbin / 1931.IX.30; 1♀ Mao’er Shan / 1962, Comprehensive Investigation Department, Ministry of Forestry coll.; 1♀ Dailing / 1971.V.22; **Liaoning**: 2♀ Qian Shan / 1987.VI.2, Jinke Li coll.; 1♀ Qingyuan / 1934.5.12; 2♂2♀, Shenyang, Qipan Shan, 2020.VII.11, Haicheng Shan coll.; 1♀, Shenyang, Qipan Shan, 2020.VII.13, Haicheng Shan coll.; 2♂, Shenyang, Qipan Shan, 2020.VII.23, Haicheng Shan coll.; 1♂1♀, Shenyang, Qipan Shan, 2020.VIII.2, Haicheng Shan coll.; 60 (♂, ♀), Shenyang, Qipan Shan, 2021.V.10–VI.13, Haicheng Shan coll.; **Hebei**: 1♂ Wuling Shan, 800 m, 1981.VI.1, Peiyu Yu coll.; 5♀ Wuling Shan, Liushuigou, 1400 m, 1981.VI.4, Peiyu Yu coll.; **Beijing**: 1♀ Xiaolongmen, Forestry Farm, elevation 1140 m, 2003.V.18, Dakang Zhou coll.; 1♀ Yanqing, Song Shan, elevation 800 m, 2003.VI.4–7, Dakang Zhou coll.; 1♀ Wuling Shan, Western Gate, host unknown / 2006.V.4, Ye Liu coll.; **Zhejiang**: 1♀ Tianmu Shan, 1936.VI.9; 2♂ Tianmu Shan, 1936.VII.23; **Fujian**: 2♀ Wuyi Shan, Nature Reserve, 670–1420 m, 2004.IV.24–5.13, Dakang Zhou coll. **Russia**: 1♂, Primorsky Krai 12 km. Chernigovka, Gribnoe / Punza / 1974.V.16 Ler. **South Korea**: 1 (NIBR), Korea (GB) Bonghwa-gun, Chunyang- myeon, Seobyeok-ri. Joong Youb Kim, 2018.V.23 / Liliocerissinica (Heyden, 1887b), Det: Jong Eun Lee, 2018.IX.19 / NIBR 0000921396.

**Figures 6–11.**
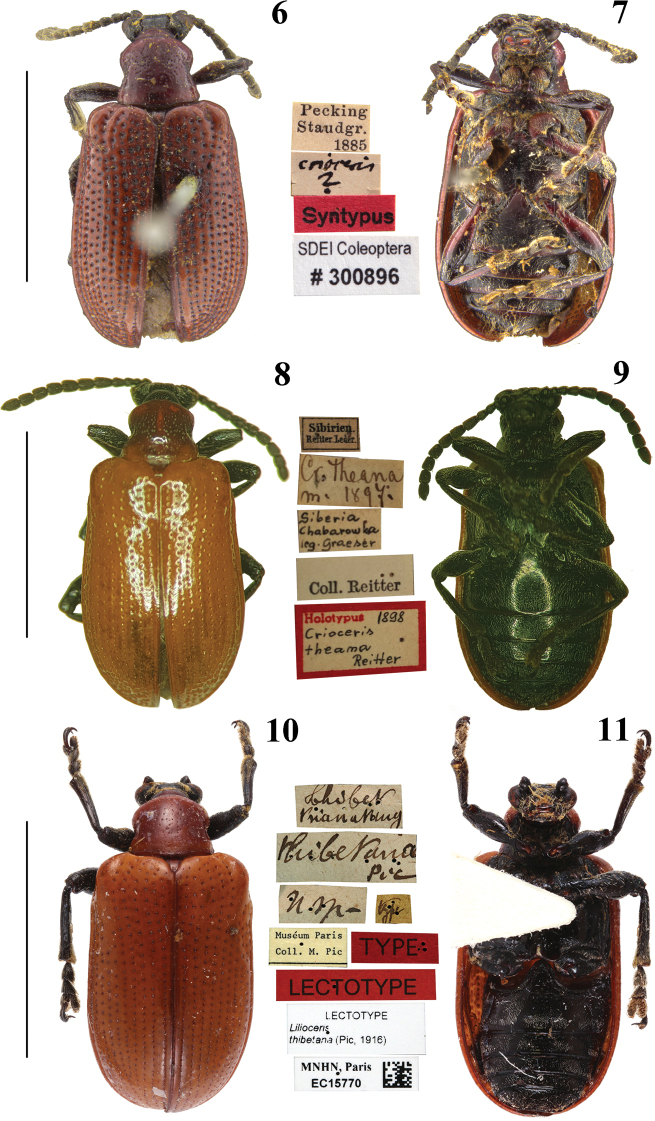
Habitus of *Lilioceris* spp. **6, 7***L.sinica*, type, China (Pecking = Beijing), photographed by Mandy Schröter **8, 9***L.theana*, holotype, Siberia, photographed by Raorao Mo **10, 11***L.thibetana*, type, China (Tibet), photographed by Antoine Mantilleri. Scale bars: 5.0 mm.

##### Diagnosis.

Femora bicolored, black with brownish red middle; pronotum disc with fine punctures; elytral punctures strong, not diminishing posteriorly, intervals convex at apical 1/4; lateral side of metasternite with a wide strip of pubescence; abdominal transverse impressions absent on sternites 2–5, sternite wholly pubescent.

##### Redescription.

BL = 7.2–8.0 mm, BW = 3.5–3.8 mm. The front part of the head, antennae, ventral surface black; occiput, pronotum, scutellum and elytra brownish red, femora bicolored, brownish red with apex black.

***Head*** (Fig. 8). HL/HW = 1.1–1.3; vertex with a deep groove in the middle, punctate and setose laterally; frontoclypeal area triangular, disc with dense punctures and setae; labrum transverse, with sparse setae; antennomeres 5–10 slightly longer than wide (Fig. 34).

***Pronotum*** (Fig. 16A). PW / HW = 0.9–1.1, PL / PW = 1.0–1.2; anterior angle slightly protruding; posterior angle not protruding; sides slightly constricted in the middle; middle of disc with fine and scattered punctures; anterior and posterior transverse impression indistinct, basal transverse groove shallow.

***Elytra*** (Fig. 16D). EL/EW = 1.4–1.5; sutural angle rounded; humeri protruding, humeral groove shallow, basal transverse impression indistinct; scutellary striole composed of 6–8 punctures; strial punctures large, not diminishing posteriorly, intervals convex at apical 1/4; epipleura raised, with a row of fine punctures laterally.

***Mesosternite pubescent***. Lateral side of metasternite with wide strip of pubescence, extending from anterior to posterior margin, 1/4 near metepisternum sparsely pubescent (Fig. 16B); metepisternum densely pubescent.

Lateral transverse impressions absent on abdominal sternites 2–5, all sternites densely pubescent (Fig. 16C).

***Legs*** slender; tibiae with dense punctures pubescence; femora with dense pubescence on dorsal surface, with sparse pubescence on ventral surface.

***Male genitalia*** (Fig. 22A–D). Median foramen occupying 1/4 length of median lobe (Fig. 22A); apex rounded (Fig. 22B); basal piece of tegmen triangular, relatively broad, lateral lobes strongly sclerotized; posterior part of dorsal sclerite in dorsal view in dorsal view widely rounded, directed laterally (Fig. 22C, D).

***Female reproductive organs*** (Fig. 28A–C). Spiculum gastrale long, Y-shaped, distal part slightly widened, apical margin rounded; ovipositor with dense setae, distal part of the ovipositor cylindrical, long, with small protuberance; spermatheca simply convoluted.

##### Host plant.

This species feeds on *Dioscoreanipponica* in Liaoning Province (Fig. 54). Adults appeared on host plants from May to September.

##### Distribution.

China (Heilongjiang, Liaoning, Jilin, Hebei, Beijing, Zhejiang, Fujian); Russia; Korea.

##### Remarks.

*Lilioceristheana* was described by Reitter (1898) from Siberia, Russia. Chûjô (1941: 453) synonymized it with *L.rugata*, and Gressitt and Kimoto (1961: 58) synonymized it with *L.sinica*. Subsequent researchers have followed Chûjô’s treatment (e.g., Warchałowski 2011; Bezděk and Schmitt 2017). According to our study of the types (Figs 8, 9), *L.theana* is a distinct species, and it clearly differs from *L.rugata* by having a wide strip of pubescence on the lateral side of the metasternite and abdominal sternites with dense pubescence (sides of metasternite and abdominal sternites nearly smooth in *L.rugata*). *Lilioceristheana* differs from *L.sinica* in the transverse impressions on abdominal sternites 2–5 absent (having clear transverse impressions on abdominal sternites 2–5 in *L.sinica*). In addition, the spiculum gastrale and spermatheca of the three species are distinctly different (Figs 25A–C, 27A–C, 28A–C).

#### ﻿ Lilioceris thibetana

Taxon classificationAnimaliaColeopteraChrysomelidae

(Pic, 1916)

77689E79-636B-5944-A0BC-000454B7FDA2

Figs 10 , 11 , 17 , 23 , 29 , 35 , 42

thibetana Pic, 1916: 18 (China, Prov. Thibet, Type / Lectotype, male). (Crioceris). Gressitt and Kimoto 1961: 59 (Lilioceris).

##### Type material examined.

1♂, type [MNHN, photo], Thibet, Trianatang / thibetana Pic / n. sp / Type / Museum Paris Coll. M. Pic / TYPE / LECTOTYPE / LECTOTYPE Lilioceristhibetana (Pic, 1916) / MNHN, Paris EC15770.

##### Other material examined.

Total 3 specimens. **China: Yunnan**: 1♂, Xishuangbanna, Meng-a, 1050–1080 m / 1958.VI.9, Shuyong Wang coll.; 1♀, Xishuangbanna, Menghai, 1200–1600 m / 1958.VII.22, Fuji Pu coll.; 1♂, Lushui, Pianma, 1750 m / 1981.V.27, Xuezhong Zhang coll. / ? Liliocerisgressitti Medvedev, Peiyu Yu det.

**Figures 12–17.**
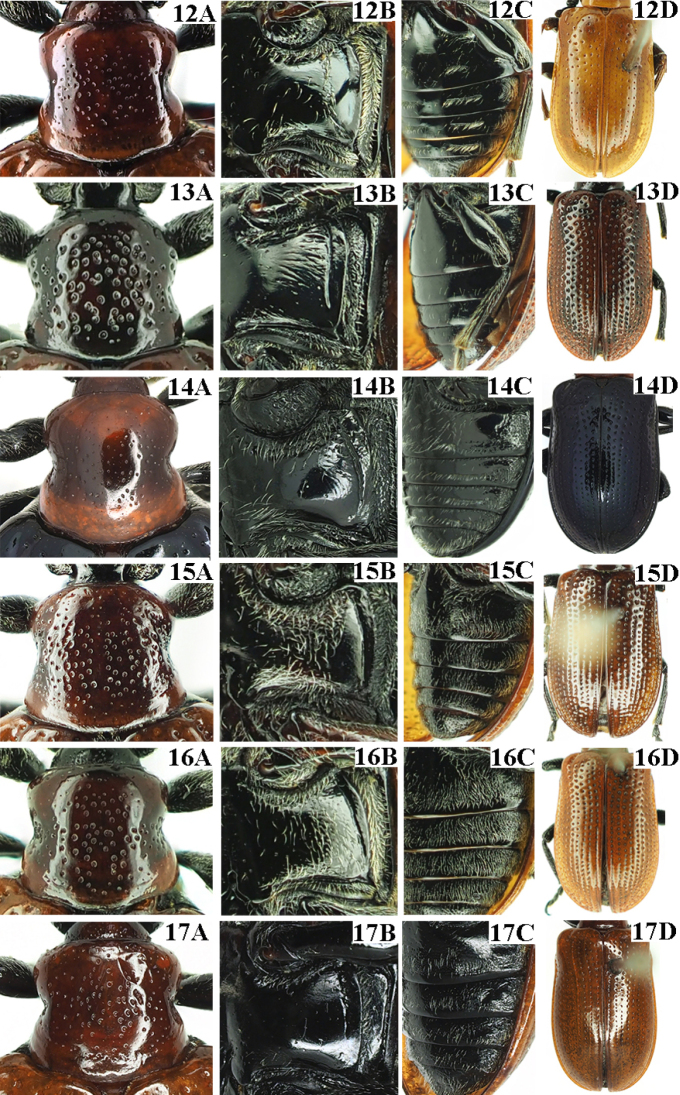
Pronotum, mesoventral disc, abdominal sternites and elytra of *Lilioceris* spp. **12***L.gressitti*, ♂, China (Yunnan: Kunming) **13***L.rugata*, ♂, Japan (Mont Takao) **14***L.sieversi*, ♂, China (Beijing)**15***L.sinica*, ♂, China (Beijing) **16***L.theana*, ♀, China (Liaoning: Shenyang) **17***L.thibetana*, ♀, China (Yunnan: Xishuangbanna) **A** pronotum **B** mesoventral disc **C** abdominal sternite **D** elytra.

##### Diagnosis.

Femora black. Pronotum disc with fine punctures; elytral punctures small, slightly diminishing or not diminishing posteriorly; metasternite almost glabrous; abdominal sternites with sparse pubescence, transverse impressions present on sternites 2–5.

##### Redescription.

BL = 6.0–7.0 mm, BW = 3.0–3.5 mm. Front part of head, antennae, ventral surface, and legs black; occiput, pronotum, scutellum, and elytra brownish red.

**Figures 18–23.**
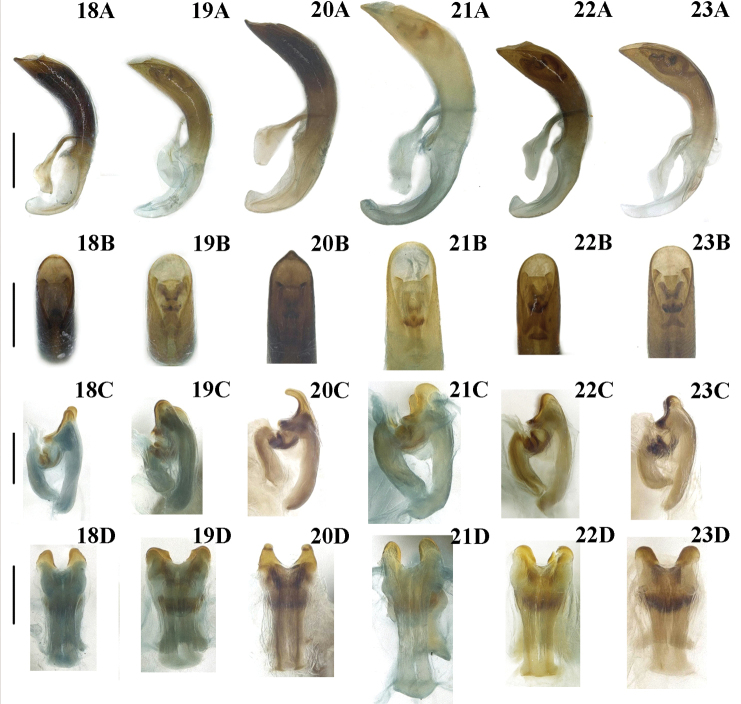
Male genitalia of *Lilioceris* spp. **18***L.gressitti*, China (Yunnan: Wuding) **19***L.rugata*, Japan **20***L.sieversi*, China (Beijing) **21***L.sinica*, China (Beijing) **22***L.theana*, China (Liaoning: Shenyang) **23***L.thibetana*, China (Yunnan: Xishuangbanna **A** aedeagus, lateral view **B** aedeagus, dorsal view **C** sclerites in internal sac, lateral view **D** dorsal sclerite, dorsal view. Scale bars: 0.5 mm (**A, B**); 0.2 mm (**C, D**).

***Head*** (Fig. 10). HL/HW = 1.1–1.2; vertex without groove in the middle, finely punctate and setose laterally; frontoclypeal area triangular, disc with sparse punctures and setae; labrum transverse, with sparse setae; antennomeres 5–10 each slightly longer than wide (Fig. 35).

***Pronotum*** (Fig. 17A). PW / HW = 1.0–1.1, PL / PW = 0.9–1.0; anterior and posterior angle not protruding; sides distinctly constricted in the middle; middle of disc with fine and scattered punctures; anterior and posterior transverse impression indistinct, basal transverse groove shallow.

**Figures 24–29.**
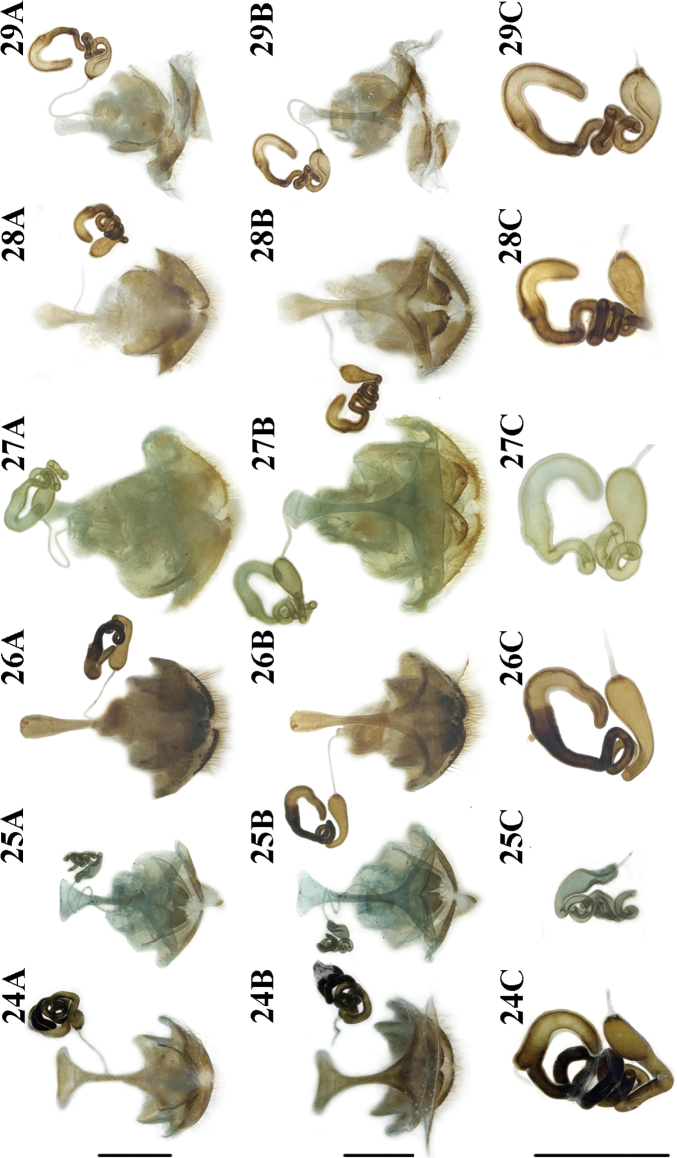
Female reproductive organs of *Lilioceris* spp. **24***L.gressitti*, China (Yunnan: Wuding) **25***L.rugata*, Japan (Tokyo) **26***L.sieversi*, China (Beijing) **27***L.sinica*, China (Beijing) **28***L.theana*, China (Liaoning: Shenyang) **29***L.thibetana*, China (Yunnan: Xishuangbanna) **A** dorsal view **B** ventral view **C** spermatheca. Scale bars: 0.5 mm.

***Elytra*** (Fig. 17D). EL/EW = 1.3–1.5; sutural angle rounded; humeri protruding, humeral groove distinct, basal transverse impression indistinct; scutellary striole composed of 5–8 punctures; strial punctures small, slightly diminishing or not diminishing posteriorly; intervals flat; epipleura raised, with row of fine punctures.

***Mesosternite pubescent***. Lateral side of the metasternite nearly smooth (Fig. 17B); metepisternum densely pubescent.

**Figures 30–41.**
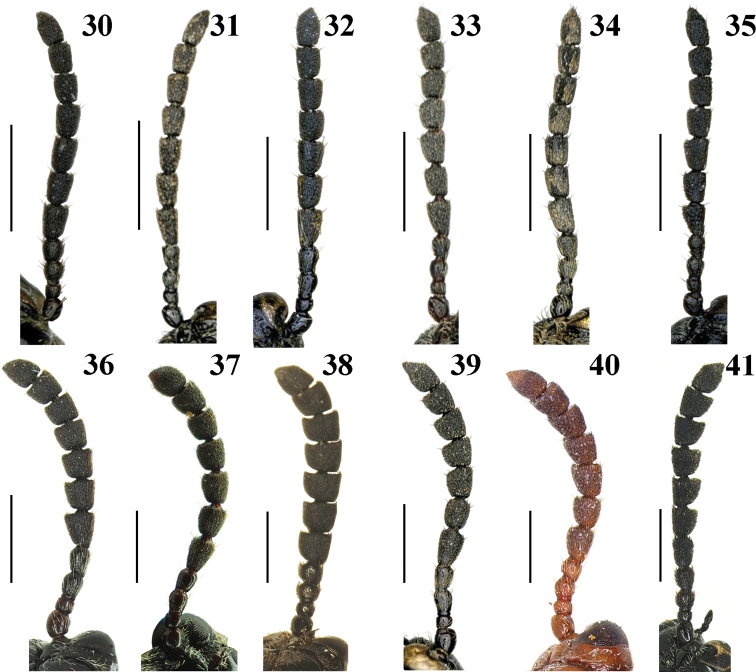
Antennae of *Lilioceris* spp. **30***L.gressitti*, ♂, China (Yunnan: Tengchong) **31***L.rugata*, ♂, Japan (Kibune: Kyoto) **32***L.sieversi*, ♀, China (Beijing) **33***L.sinica*, ♂, China (Anhui: Yuexi) **34***L.theana*, ♀, China (Liaoning: Shenyang) **35***L.thibetana*, ♂, China (Yunnan: Lushui) **36***L.cheni*, ♂, China (Guangdong: Shixing) **37***L.egena*, ♂, China (Tibet: Mêdog) **38***L.impressa*, ♂, China (Yunnan: Gongshan) **39***L.laosensis*, ♂, China (Tibet: Mêdog) **40***L.malabarica*, ♂, India (Mahe: Malabar) **41***L.yunnana*, ♂, China (Yunnan: Tengchong). Scale bars: 1.0 mm.

Abdominal sternites with sparse pubescence; lateral transverse impressions present on sternites 2–5, area outside the impression densely pubescent (Fig. 17C).

**Figure 42.**
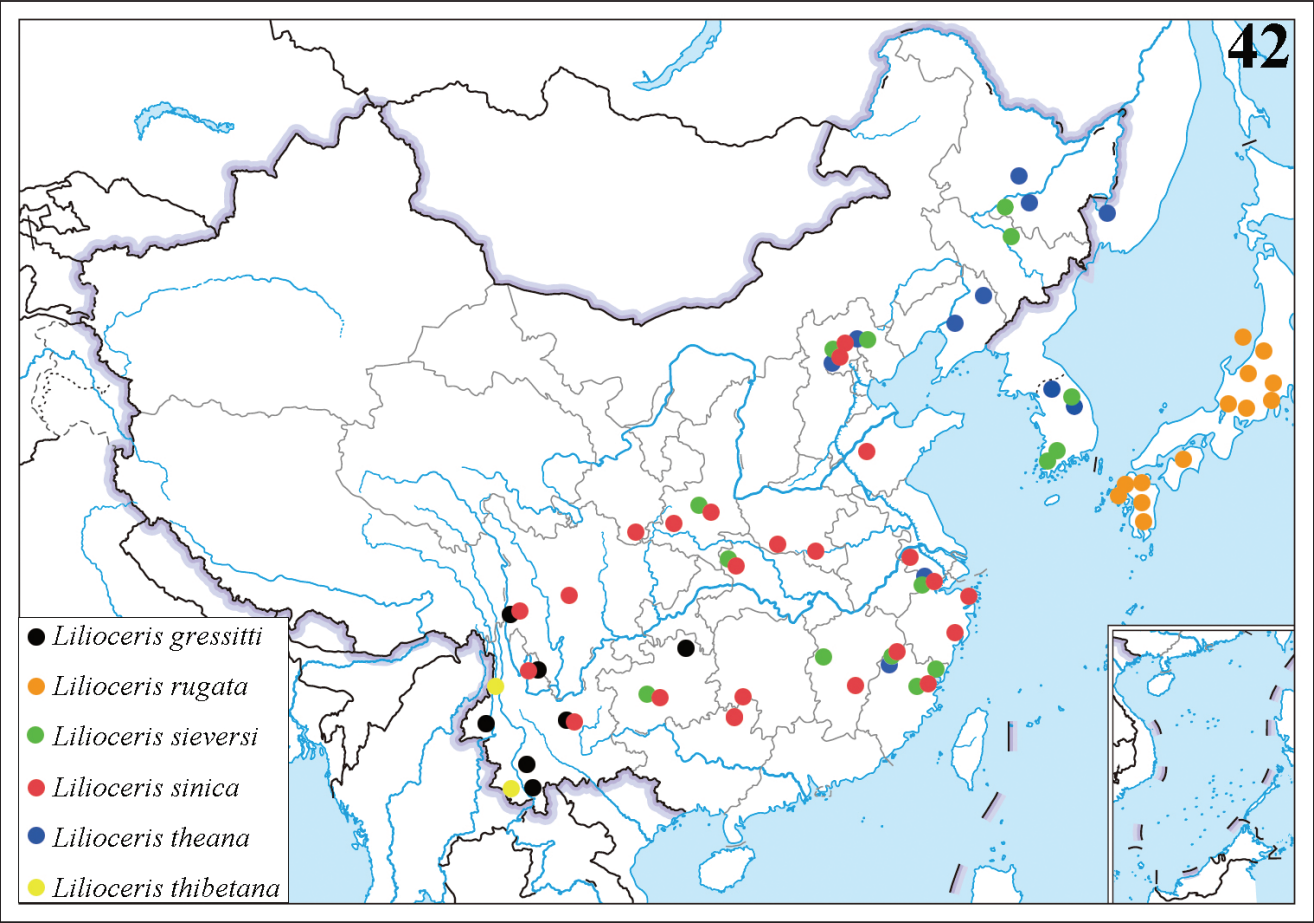
Distribution map of *Lilioceris* spp. (*L.sinica* in Korea and *L.thibetana* in Tibet are not marked because of lack of precise locality data).

***Legs*** slender; tibiae with dense punctures and pubescence; femora with dense pubescence on dorsal surface, with sparse pubescence on ventral surface.

***Male genitalia*** (Fig. 23A–D). Median foramen occupying 1/5 length of median lobe (Fig. 23A); apex rounded (Fig. 23B); basal piece of tegmen triangular, lateral lobes slightly sclerotized; posterior part of dorsal sclerite in dorsal view in dorsal view widely rounded, directed laterally (Fig. 23C, D).

***Female reproductive organs*** (Fig. 29A–C). Spiculum gastrale short, Y-shaped, distal part slightly widened, apical margin rounded (it was broken during dissection, Fig. 29A, B); ovipositor with dense setae, distal part of the ovipositor cylindrical, short, with small protuberance; spermatheca greatly convoluted.

**Figures 43, 44.**
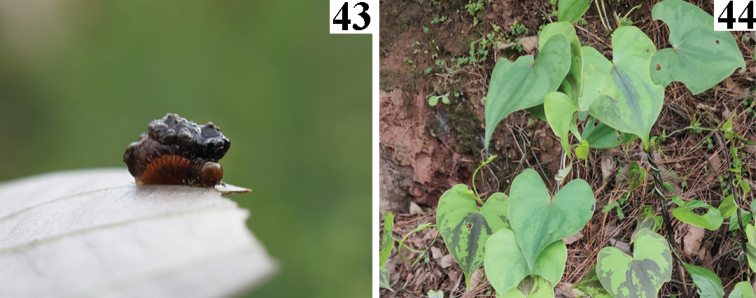
*Liliocerisgressitti* in China (Yunnan: Wuding), 2021.VII.11, photographed by YX **43** larva **44** host plant, *Dioscorea* sp.

##### Distribution.

China (Tibet, Yunnan).

##### Host plant and habitat.

Unknown.

##### Remarks.

In original labels, the type locality is ‘Thibet, Trianatang’. There are at least three villages with similar pronunciation to Trianatang, first village ‘Qiunatong’ is in Gongshan county, northwestern Yunnan (28.09655°N, 98.57368°E, 1816 m), very close to Tibet; the second village ‘Qunatang’ is in Zayü county, Tibet (28.33884°N, 98.58602°E, 2460 m), and the third village ‘Qunatang’ is in Mêdog county, Tibet (29.46423°N, 95.74406°E, 2084 m). They are not far from each other, and all are possibilities to be the type locality of ‘Trianatang’.

**Figures 45, 46.**
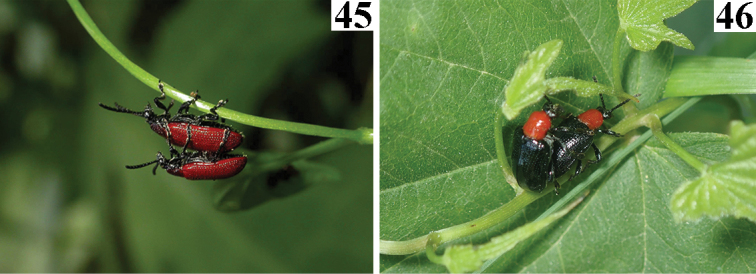
*Lilioceris* spp. **45***Liliocerisrugata* in Japan, 2003.V.9, photographed by Masakazu Hayashi **46***Liliocerissieversi* in China (Beijing), 2021.VI.12, photographed by Meiying Lin.

*Lilioceristhibetana* was formerly placed in the *impressa* group (Tishechkin et al. 2011), probably due to its similarity with *Liliocerismalabarica* as stated in original description by Pic (1916). In the holotype, the antennae are missing, so it is difficult to determine whether it belongs to the *impressa* group or the *sinica* group based on the antennae. Fortunately, we have three specimens from Yunnan which fit well with the type in body size, body color, punctures on pronotum and elytra, pubescence on metasternites and abdominal sternites, and in the shape of the aedeagus (compared with the illustration of Tishechkin et al. 2011: fig. 29). However, their antennomeres 5–10 are all cylindrical so we moved *L.thibetana* into the *sinica* group.

**Figures 47–50.**
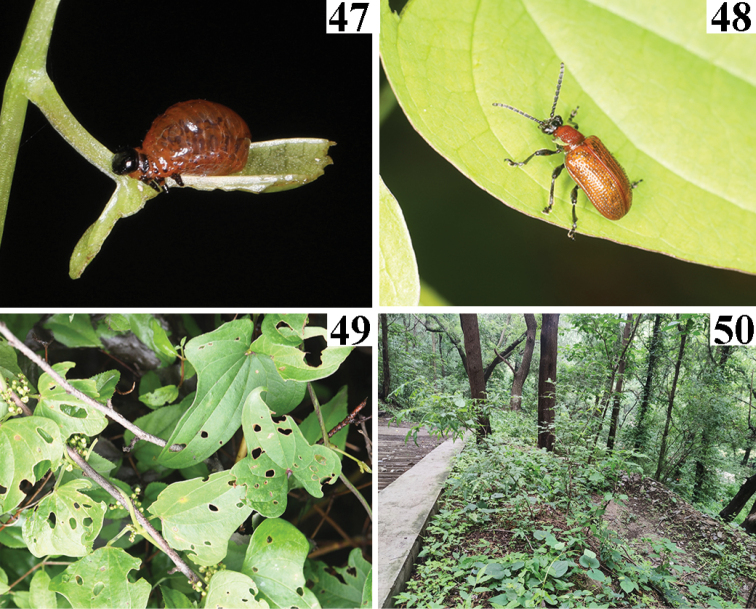
*Liliocerissinica* in China (Beijing), 2021.VII.16 **47** larva **48** adult **49** host plant, *Dioscorea* sp. **50** Habitat **47, 48** photographed by HBL. **49, 50** photographed by YX.

This species is similar to *Liliocerisgressitti*, but differs by having the metaventral disc nearly smooth (in *L.gressitti*, the metaventral disc has a narrow pubescent strip). Furthermore, in *L.thibetana*, the spiculum gastrale is Y-shaped, slightly wider in the distal part, and the apical margin is rounded, while in *L.gressitti*, the spiculum gastrale is X-shaped, strongly widened in the distal part, and the apical margin is straight.

**Figures 51–54.**
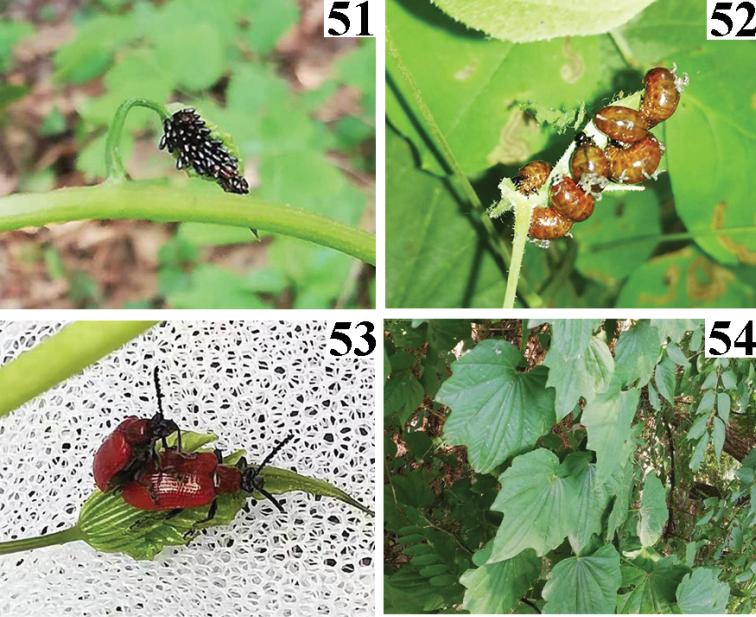
Biology of *Lilioceristheana*. China (Liaoning: Shenyang), 2021.V.23, Photographed by Haicheng Shan **51** eggs **52** larvae **53** adults **54** host plant, *Dioscoreanipponica*.

## Supplementary Material

XML Treatment for Lilioceris gressitti

XML Treatment for Lilioceris rugata

XML Treatment for Lilioceris sieversi

XML Treatment for Lilioceris sinica

XML Treatment for Lilioceris theana

XML Treatment for Lilioceris thibetana
